# A corpus of GA4GH phenopackets: Case-level phenotyping for genomic diagnostics and discovery

**DOI:** 10.1016/j.xhgg.2024.100371

**Published:** 2024-10-10

**Authors:** Daniel Danis, Michael J. Bamshad, Yasemin Bridges, Andrés Caballero-Oteyza, Pilar Cacheiro, Leigh C. Carmody, Leonardo Chimirri, Jessica X. Chong, Ben Coleman, Raymond Dalgleish, Peter J. Freeman, Adam S.L. Graefe, Tudor Groza, Peter Hansen, Julius O.B. Jacobsen, Adam Klocperk, Maaike Kusters, Markus S. Ladewig, Allison J. Marcello, Teresa Mattina, Christopher J. Mungall, Monica C. Munoz-Torres, Justin T. Reese, Filip Rehburg, Bárbara C.S. Reis, Catharina Schuetz, Damian Smedley, Timmy Strauss, Jagadish Chandrabose Sundaramurthi, Sylvia Thun, Kyran Wissink, John F. Wagstaff, David Zocche, Melissa A. Haendel, Peter N. Robinson

**Affiliations:** 1 Berlin Institute of Health at Charité – Universitätsmedizin Berlin, Berlin, Germany; 2 The Jackson Laboratory for Genomic Medicine, 10 Discovery Drive, Farmington CT 06032, USA; 3 Department of Pediatrics, Division of Genetic Medicine, University of Washington, 1959 NE Pacific Street, Box 357371, Seattle, WA 98195, USA; 4 Brotman-Baty Institute for Precision Medicine, 1959 NE Pacific Street, Box 357657, Seattle, WA 98195, USA; 5 Department of Pediatrics, Division of Genetic Medicine, Seattle Children’s Hospital, Seattle, WA 98195, USA; 6 William Harvey Research Institute, Queen Mary University of London, London, UK; 7 Clinic for Immunology and Rheumatology, Hanover Medical School, Hanover, Germany; 8 RESiST-Cluster of Excellence 2155, Hanover Medical School, Hanover, Germany; 9 Department of Genetics, Genomics and Cancer Sciences, University of Leicester, Leicester, UK; 10 Division of Informatics, Imaging and Data Science, The University of Manchester, Manchester, UK; 11 Rare Care Centre, Perth Children’s Hospital, Nedlands, WA 6009, Australia; 12 SingHealth Duke-NUS Institute of Precision Medicine, 5 Hospital Drive Level 9, Singapore 169609, Singapore; 13 Telethon Kids Institute, Nedlands, WA 6009, Australia; 14 Department of Immunology, 2nd Faculty of Medicine, Charles University and University Hospital in Motol, Prague, Czech Republic; 15 Department of Paediatric Immunology, Great Ormond Street Hospital for Children NHS Foundation Trust, London, UK; 16 University College London Institute of Child Health, London, UK; 17 Department of Ophthalmology, University Clinic Marburg - Campus Fulda, Fulda, Germany; 18 Medica Genetics University of Catania Italy, Catania, Italy; 19 Morgagni Foundation and Clinic, Catania, Italy; 20 Division of Environmental Genomics and Systems Biology, Lawrence Berkeley National Laboratory, Berkeley, CA, USA; 21 Department of Biomedical Informatics, University of Colorado Anschutz Medical Campus, Aurora, CO, USA; 22 Department of Allergy and Immunology, National Institute of Women’s, Children’s and Adolescents' Health Fernandes Figueira, Rio de Janeiro, Brazil; 23 High Complexity Laboratory, National Institute of Women’s, Children’s and Adolescents' Health Fernandes Figueira, Rio de Janeiro, Brazil; 24 Department of Pediatrics, Faculty of Medicine and University Hospital Carl Gustav Carus, Technische Universität Dresden, Dresden, Germany; 25 University Center for Rare Diseases, Faculty of Medicine and University Hospital Carl Gustav Carus, Technische Universität Dresden, Dresden, Germany; 26 Utrecht University, Utrecht, the Netherlands; 27 North West Thames Regional Genetics Service, Northwick Park & St Mark’s Hospitals, London, UK; 28 University of North Carolina at Chapel Hill, Chapel Hill, NC, USA; 29 ELLIS-European Laboratory for Learning and Intelligent Systems; 30 German Center for Child and Adolescent Health (DZKJ), partner site Leipzig/Dresden, Dresden, Germany

**Keywords:** human phenotype ontology, global alliance for genomics and health, phenopacket schema

## Abstract

The Global Alliance for Genomics and Health (GA4GH) Phenopacket Schema was released in 2022 and approved by ISO as a standard for sharing clinical and genomic information about an individual, including phenotypic descriptions, numerical measurements, genetic information, diagnoses, and treatments. A phenopacket can be used as an input file for software that supports phenotype-driven genomic diagnostics and for algorithms that facilitate patient classification and stratification for identifying new diseases and treatments. There has been a great need for a collection of phenopackets to test software pipelines and algorithms. Here, we present Phenopacket Store. Phenopacket Store v.0.1.19 includes 6,668 phenopackets representing 475 Mendelian and chromosomal diseases associated with 423 genes and 3,834 unique pathogenic alleles curated from 959 different publications. This represents the first large-scale collection of case-level, standardized phenotypic information derived from case reports in the literature with detailed descriptions of the clinical data and will be useful for many purposes, including the development and testing of software for prioritizing genes and diseases in diagnostic genomics, machine learning analysis of clinical phenotype data, patient stratification, and genotype-phenotype correlations. This corpus also provides best-practice examples for curating literature-derived data using the GA4GH Phenopacket Schema.

## Teaser

Phenopacket Store includes 6,058 GA4GH phenopackets representing 469 Mendelian and chromosomal diseases, representing the first large-scale collection of case-level, standardized phenotypic information derived from published case reports, and will be useful for many purposes, including the development and testing of software to support translation research.

## Main text

Over 10,000 rare diseases (RDs) have been identified to date,^1^ collectively affecting between 3.5% and 8% of the population,^2^ yet many patients experience a long diagnostic odyssey of 5–7 years.^1^^,^^3^ Previously, each of the numerous software packages that support phenotype-driven genomic diagnostics for RDs has used bespoke input formats for phenotypic data and information about the pedigree. The Phenopacket Schema provides a standard input format for such tools that will simplify computational analysis pipelines.

Ontologies are systematic representations of knowledge that can be used to capture medical phenotype data by providing concepts (terms) from a knowledge domain and additionally specifying formal semantic relations between the concepts. Ontologies enable precise patient classification by supporting the integration and analysis of large amounts of heterogeneous data.^4^ The Human Phenotype Ontology (HPO), developed by the Monarch Initiative,^5^ is widely used in human genetics and other fields that care for individuals with RDs^6^ and is also increasingly being used in other settings, such as electronic health records (EHRs).^7^^,^^8^ HPO terms represent phenotypic features such as signs, symptoms, and laboratory and imaging findings. However, the HPO itself does not specify how HPO terms and data should be arranged to record and exchange such information along with genomic data. To address this, in the context of the Global Alliance for Genomics and Health (GA4GH), we developed the Phenopacket Schema, a standard for sharing disease and phenotype information. A phenopacket is a computational representation of an individual person or biosample, linking that individual to phenotypic descriptions, genetic information, diagnoses, and treatments.^9^^,^^10^

The Phenopacket Schema allows clinical data (phenotypic attributes, measurements, treatments, and other medical actions) from individual patients to be compared and shared broadly, in contrast to the sensitive clinical data found within EHRs and other contexts. Such comparisons can aid in diagnosis and facilitate patient classification and stratification for identifying new diseases and treatments.^11^ The Phenopacket Schema is designed to support interoperability between people, organizations, and systems to advance the worldwide effort to address human disease and biological understanding. These partners include clinical laboratories, authors, journals, clinicians, data repositories, patient registries, EHR systems, and knowledge bases. The Phenopacket Schema does not model -omics data in detail but does enable users to link a phenopacket to files representing data from high-throughput screening techniques or to denote individual variants in several formats.^11^ The Phenopacket Schema integrates a version of the GA4GH Variant Representation Specification and is designed to be interoperable with other GA4GH standards, including those for pedigree data.^12^

The Phenopacket Schema aims to represent data from different sources, including data from EHRs, research studies, data entry tools, or published case reports, in a consistent and computable format to enable the sharing and integration of structured clinical data. The core principles of the schema include composability, traceability (data provenance), the FAIR (findable, accessible, interoperable, and reusable) principles, and computability.^13^ Multiple upstream data collection and management tools already support exporting patient profiles as phenopackets for downstream analysis and data sharing, including PhenoTips,^14^ RD-Connect Genome-Phenome Analysis Platform (GPAP),^15^ Patient Archive in Australia, and IRUD Exchange in Japan.^16^ PhenoTips can generate phenopackets from patient or family records through a user interface or REST APIs and includes de-identified demographic data, clinical phenotype, diagnoses, curated genetic findings, and pedigree data.^17^ Exomiser,^18^^,^^19^ LIRICAL,^20^ SvAnna,^21^ Phen2Gene,^22^ and CADA^23^ already accept phenotype data in Phenopacket format. Projects such as the EU-funded Solve-RD and the European Joint Programme on Rare Diseases (EJP-RD) can generate phenopackets for the data included in GPAP, which aims to facilitate diagnosis and novel gene discovery for clinical researchers.^24^ Phenopackets are used in Solve-RD to share phenotypic and other relevant clinical or genetic information (e.g., candidate or causative variants) between the consortium members and are also deposited along the genomics data at the European Genome-Phenome Archive (EGA) for long-term archival and controlled access. Besides being a successful instrument for data import/export between the project’s databases, phenopackets represent a computational model of a patient trajectory that has proved to be useful for data analysis, such as clustering patients based on their phenotypic similarity.^25^

There is a need for a collection of phenopackets to test the software pipelines and algorithms that work on individual rare and genetic disease patient cases. In this work, we have created Phenopacket Store, a collection of 6,668 phenopackets with clinical data from individuals with one of 475 Mendelian and chromosomal diseases. We developed pyphetools, a Python package with functionality to streamline the creation of phenopackets from tabular data often found in the medical literature. We selected publications for curation from the human genetics literature to represent a broad range of diseases. Publications were considered if they presented individual-level data about one or more individuals affected by a given disease. Publications were not included if they provided only aggregate or summary-level information. For instance, if 7/12 patients in some cohort were reported to have scoliosis and 3/12 to have pes planus, but no information was provided about the specific features that each of the individuals in the cohort had, then the publication would not be a candidate for inclusion in Phenopacket Store. A typical table contains information about patients in rows and one column for each data item (age of onset, sex, genetic variants, phenotypic features, etc.). For publications that do not contain such tables, pyphetools offers various helper functions that assist with manual curation and filling of an Excel template from which phenopackets can then be created. The Phenopacket Schema is a model that can be stored in many formats. We recommend JSON and have stored each phenopacket in this repository as a JSON file.

**Figure 1:**
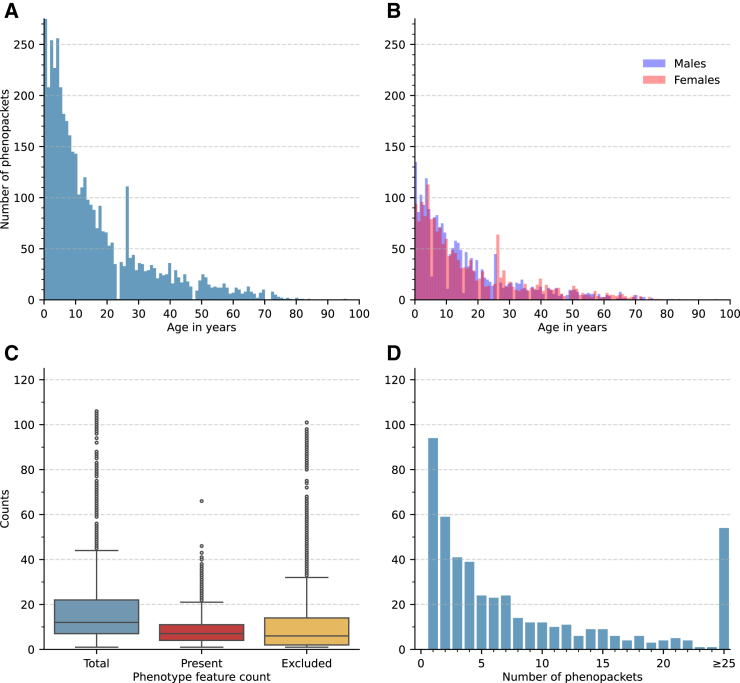
Phenopacket Store summary characteristics (A) A histogram with distribution of ages of last examination. (B) The histogram of age of last examination partitioned by sex. (C) Distribution of HPO term counts per phenopacket. The boxplots show the counts of the HPO terms present in the phenopacket, the terms that were specifically excluded, and the total HPO count (present + excluded). The horizontal line of each box indicates the median term count, box borders indicate positions of the 1st and 3rd quartiles, the whiskers indicate 1.5 times the interquartile range, and the circles represent the term counts beyond the interquartile range. (D) The number of diseases for which the indicated number of phenopackets is available.

One of the goals of the Phenopacket Store project is to provide a collection of best-practice phenopackets for rare genetic diseases that will enable software developers to test program code and develop novel algorithms. We have curated a wide range of rare diseases including cohorts ranging from 1 to 463 individuals. Phenopacket Store comprises phenopackets representing 6,668 individuals diagnosed with 475 diseases. 75.6% of the 6,668 phenopackets had the sex of the individuals specified; of these, 52.8% were males and 47.2% were females. The individuals are partitioned into cohorts based on the genes harboring the disease-causing mutations. There are 423 gene cohorts in total. Of these, 25 genes were associated with two Mendelian diseases, and 11 genes were associated with more than two diseases. The maximum number of diseases associated with a single gene was 6 diseases in the case of *FBN1.* On average, 14.0 individuals were curated per disease. 3,834 distinct variants are included, and the information was derived from 959 different publications. In total, 3,292 distinct HPO terms were used, and the cohorts include, on average, 127.2 present and 146.0 excluded HPO terms (Table 1). On the case report level, the individuals are annotated with 8.2 present and 11.8 excluded HPO terms on average (Figure 1).

**Table 1:** The summary characteristics of 423 cohorts presented in v.0.1.19 of Phenopacket Store

	Phenopacket count		Diseases	Genes	Unique Alleles	Publications	Phenotypic feature count		
	Per cohort	Per disease					Present	Excluded	Total
Mean	15.8	14.0	1.1	1	9.2	2.29	127.2	146.0	273.2
Median	4	5	1	1	3	1	37	17	57
Minimum	1	1	1	1	1	1	2	0	2
Maximum	463	463	6 (*FBN1*)	1	264	28	4130	4844	5928
Total	6668		475	423	3834	959	53,693	615,98	115,291

The pyphetools library contains extensive quality-control code to prevent format errors. We additionally validate each of the phenopackets using the Java command-line application called phenopacket-tools.^11^ We have created the phenopackets with the following rules and assumptions.

### Phenopacket and individual IDs

In the GA4GH Phenopacket Schema, both the phenopacket and the individual (patient) have identifiers (IDs). We have used the IDs in the original publications for the individual ID. If no ID was provided, then we used the word “individual.” Note that the individual ID must be distinct for all individuals described in any publication. For the phenopacket ID, we prepended the PubMed ID. For instance, in a publication about variants in the *VRK1* gene,^26^ an individual with the ID BAB3022 was described. We use this for the individual ID, and for the phenopacket ID, we use PMID_24126608_BAB3022. The Phenopacket Schema does not require PubMed IDs, but for this repository, we are only including published care reports with a PubMed ID. We ensured that a unique ID is used for each individual described in a publication so that the combination of PMID and ID is unique across all phenopackets in Phenopacket Store. In some cases, a single individual has been published several times with different IDs (see, for instance, individual #00318253 in the Leiden Open Variation Database^27^). It is outside the scope of the Phenopacket Schema to address the issue of duplication, but we recommend that curators be aware of this potential problem and take measures not to create multiple phenopackets that represent the same individual.

### Age of onset and age at last examination

Wherever possible, the age of onset was curated from the original publication (i.e., the age of the first manifestation of the disease). Additionally, the age at the latest examination was curated. Some phenopackets additionally have information about the age of death (if applicable).

Where available, the age of onset of individual phenotypic features was also recorded. However, this information is not uniformly provided in the medical literature, and only 4,913 (4.3%) of the total of 115,291 phenotypic feature annotations had an associated age of onset.

### Disease diagnosis

**Figure 2:**
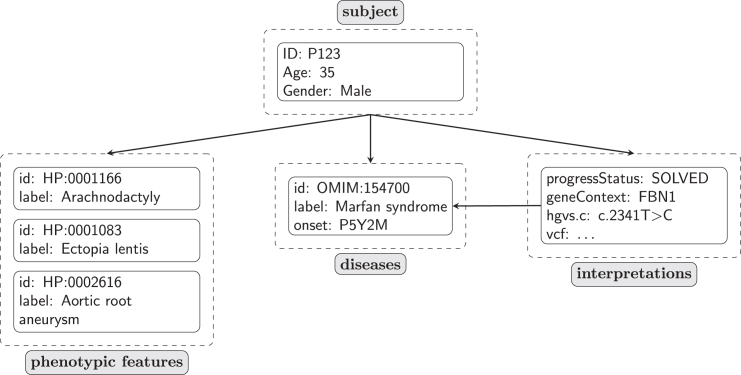
Schematic visualization of a phenopacket In this simplified representation, the major elements of the Phenopacket Schema used for the phenopackets in this collection are shown. The subject of the phenopacket is represented using the individual element, which allows the (anonymous) identifier, age at last examination, and sex to be specified. Each subject can have an arbitrary number of phenotypic features, which comprise an HPO term and, optionally, information about the age of onset of the feature. The subject can have an arbitrary number of diseases, but for the phenopackets contained in this collection, each subject has one disease. The subject can have an arbitrary number of interpretations, which must refer to a disease in the disease list. In this example, a pathogenic variant in the *FBN1* gene is interpreted to be causal for Marfan syndrome. Note that the Phenopacket Schema can additionally represent treatments, numerical measurements, and other clinical data. For a more detailed illustration, see the original publication.^9^

We encode the disease diagnosis in the top-level list of disease elements. The Phenopacket Schema does not specify which disease terminology should be used; use of the Online Mendelian Inheritance in Man (OMIM) IDs^28^ or Mondo Disease Ontology IDs are recommended.^29^ The age of onset, the age of manifestation of the first sign or symptom of a disease, is encoded as a part of the disease element. Because the current collection of phenopackets is focused on representing published case reports with genetic diagnoses, the disease is also recorded in the diagnosis attribute of the top-level interpretation element. The disease ID recorded in the diagnosis must match an ID of one of the diseases in the top-level disease list or an error will be recorded. Note that for other purposes, the top-level list of disease elements could record additional diseases or could use a Mondo term such as nonsyndromic genetic hearing loss (MONDO:0019497) to represent the clinical diagnosis made before genetic testing. We have not provided these candidate diagnoses in this collection of phenopackets because, in general, the information is not available in the published clinical case reports. As of the current version of Phenopacket Store, the phenopackets use the subject, phenotypic features, disease, and interpretation top-level elements; other elements, such as medical actions and measurements, are not used, primarily because information available in the published literature is rarely sufficient to capture this kind of information. Figure 2 provides a simplified overview of the internal structure of a single phenopacket entry.

### Interpretations

A phenopacket can contain one or more interpretation elements that specify interpretations of genomic findings. For Phenopacket Store, we have included published case reports that reported variants deemed to be causal. The phenopackets in Phenopacket Store currently rely on an implementation of the GA4GH VRSATILE standard.^30^ The VariationDescriptor class contains a computational representation of HGNC gene IDs, HGVS descriptions, gene symbols, and variant zygosity. The medical literature contains many case reports in which structural variants, defined here as variants that are at least 50 nt in size but may extend to hundreds of thousands or millions of nucleotides, are represented only by a qualitative description. For instance, in a report about *SLC9A3* variants in congenital secretory sodium diarrhea 8 (OMIM: 616868), chromosomal microarray analysis revealed a heterozygous, paternally inherited 1.383 Mb deletion on chromosome 5p15.33 encompassing *SLC9A3* in patient 1. This variant was reported as “gene deletion” in table 2 of the publication,^31^ which is how the variant is represented in the phenopacket we created for Phenopacket Store. Software that uses phenopackets from this collection should be aware of this convention (see Figures S1 and S2 for examples).

Increasing the volume of computable data across a diversity of systems will support global computational disease analysis by integrating genotype, phenotype, and other multi-modal data for precision health applications. GA4GH phenopackets can be generated from a variety of source data and used for many different kinds of analysis. Phenopackets intend to make data “analysis ready” or “AI ready” so that software tools can perform various analytics tasks or queries across collections of phenopackets without extensive data transformations prior to the computational logic.

The Phenopacket Schema was designed to support a number of use cases in a range of fields including RD diagnostics, biobanking, oncology, and EHR integration. Here, we have created a substantial collection of phenopackets representing individuals diagnosed with a rare genetic disease. The collection is intended to be used by bioinformaticians and other analysts to develop and test software; for instance, the performance of a genomic diagnostic software could be tested by simulating cases using the phenopackets by spiking the causal variants reported in the phenopackets into VCF files that are representative of the population being tested. The collection also provides examples of best practices in creating phenopackets for databases or to accompany manuscripts describing case reports or cohorts of individuals with a RD. Additionally, the Monarch Initiative is currently updating its HPO annotation pipeline to use phenopackets in addition to the HPO annotations file (phenotype.hpoa).^6^

The phenopackets in Phenopacket Store represent the first large-scale collection derived from case reports in the literature with detailed descriptions of the clinical data. They will be useful for many purposes, including the development and testing of software for prioritizing genes and diseases in diagnostic genomics, machine learning analysis of clinical phenotype data, patient stratification, and genotype-phenotype correlations. They also provide a set of best-practices examples for curating literature-derived data using the GA4GH Phenopacket Schema. Genomic data will become ever more important in translational research and clinical care in the coming years and decades. The Phenopacket Schema represents a standard for capturing clinical data and integrating it with genomic data that will help to obtain the maximal utility of these data for understanding disease and developing precision medicine approaches to therapy.

## Data and code availability

Phenopacket Store is available at https://github.com/monarch-initiative/phenopacket-store under a BSD3 open-source license. The phenopackets generated with the Phenopacket Store code are available under the “releases” tab of the repository. v.0.1.19 was presented in this manuscript. Starting with v.0.1.16, each release of Phenopacket Store has additionally been made available on Zenodo (see web resources).

Phenopacket Store makes use of the pyphetools library to create phenopackets. Pyphetools is a Python library and is available at https://github.com/monarch-initiative/pyphetools under an MIT license. v.0.9.95 was current at the time of this writing. Pyphetools is additionally available at the Python Package Index (pypi) at https://pypi.org/project/pyphetools/. The Phenopacket Store Toolkit is a Python package available under a BSD3 license to simplify using the Phenopacket Store data in the downstream applications.

## Acknowledgments

Research reported in this publication was supported by the National Human Genome Research Institute (10.13039/100000051NHGRI) at the National Institutes of Health (10.13039/100000002NIH) under award nos. 1RM1HG010860 and 5U24HG011449, by the National Institute of Child Health and Human Development (10.13039/100000071NICHD) at the 10.13039/100000002NIH under award no. 5R01HD103805, and by the Director, Office of Science, Office of Basic Energy Sciences of the U.S. Department of Energy Contract No. DE-AC02-05CH11231. J.X.C. and A.J.M. were supported by 1R35HG011297 (NHGRI) and M.J.B. and J.X.C. were supported by U01HG011744 (NHGRI). P.N.R. received additional support from the 10.13039/100005156Alexander von Humboldt Foundation.

## Author contributions

D.D., J.T.R., T.G., J.O.B.J., F.R., D.S., P.N.R.: Python code; R.D., P.J.R., J.F.W.: Variant validator integration; M.J.B., Y.B., A.C.-O., P.C., L.C.C., L.C., J.X.C., B.C., A.S.L.G., P.H., A.K., M.K., M.S.L., A.J.M., T.M., B.C.S.R., C.S., T.S., J.C.S., K.W., D.Z., P.N.R.: Biocuration; C.J.M., M.C.M.T., D.S., S.T., M.A.H.: Supervision; D.D., P.N.R.: Drafting original manuscript; D.D., J.T.R., C.J.M., D.S., M.A.H., P.N.R.: Revision. All authors read and approved the final manuscript.

## Declaration of interests

M.A.H. is a founder of Alamya Health. M.J.B. and J.X.C. are the Editor-in-Chief and Deputy Editor of HGG Advances, respectively, and were recused from the editorial handling of this manuscript.

## Web resources

GA4GH Phenopacket Schema, https://github.com/phenopackets/phenopacket-schema
Human Phenotype Ontology, https://hpo.jax.org/
Pyphetools, https://github.com/monarch-initiative/pyphetools
Phenopacket Store repository, https://github.com/monarch-initiative/phenopacket-store
Phenopacket Store repository 0.1.19, https://doi.org/10.5281/zenodo.13361405
Phenopacket Store Toolkit, https://github.com/monarch-initiative/phenopacket-store-toolkit

## Supplemental information

Document S1. Figures S1 and S2

Document S2. Article plus supplemental information
