# Supplementary material for: A corpus of GA4GH phenopackets: Case-level phenotyping for genomic diagnostics and discovery
Source: HGG Adv. 2024 Oct 10;6(1):100371. doi: 10.1016/j.xhgg.2024.100371 (PMC11564936; doi:10.1016/j.xhgg.2024.100371)
Supplement: Document S1. Figures S1 and S2 [file mmc1.pdf]

HGGA, Volume *6*

Supplemental information

A corpus of GA4GH phenopackets: Case-level phenotyping for genomic diagnostics and discovery

Daniel Danis, Michael J. Bamshad, Yasemin Bridges, Andrés Caballero-Oteyza, Pilar Cacheiro, Leigh C. Carmody, Leonardo Chimirri, Jessica X. Chong, Ben Coleman, Raymond Dalgleish, Peter J. Freeman, Adam S.L. Graefe, Tudor Groza, Peter Hansen, Julius O.B. Jacobsen, Adam Klocperk, Maaike Kusters, Markus S. Ladewig, Allison J. Marcello, Teresa Mattina, Christopher J. Mungall, Monica C. Munoz-Torres, Justin T. Reese, Filip Rehburg, Bárbara C.S. Reis, Catharina Schuetz, Damian Smedley, Timmy Strauss, Jagadish Chandrabose Sundaramurthi, Sylvia Thun, Kyran Wissink, John F. Wagstaff, David Zocche, Melissa A. Haendel, and Peter N. Robinson

```
"interpretations": [
  {
    "id": "propositus",
    "progressStatus": "SOLVED",
    "diagnosis": {
      "disease": {
        "id": "OMIM:148820",
        "label": "Waardenburg syndrome, type 3"
      },
      "genomicInterpretations": [
        {
          "subjectOrBiosampleId": "propositus",
          "interpretationStatus": "CAUSATIVE",
          "variantInterpretation": {
            "variationDescriptor": {
              "id": "var_glyvibsLWJwJucNcxQaSJJoYN",
              "geneContext": {
                "valueId": "HGNC:8617",
                "symbol": "PAX3"
              },
              "expressions": [
                {
                  "syntax": "hgvs.c",
                  "value": "NM_181458.4:c.251C>T"
                },
                {
                  "syntax": "hgvs.g",
                  "value": "NC_000002.12:g.222297048G>A"
                }
              ],
              "vcfRecord": {
                "genomeAssembly": "hg38",
                "chrom": "chr2",
                "pos": "222297048",
                "ref": "G",
                "alt": "A"
              },
              "moleculeContext": "genomic",
              "allelicState": {
                "id": "GENO:0000136",
                "label": "homozygous"
              }
            }
          }
        }
      ]
    }
  }
],
```


Figure S1: Representation of a single-nucleotide variant deemed to be causative for Waardenburg syndrome type 3 in an individual with the subject identifier "propositus". The pyphetools library uses the application programmer's interface (API) of VariantValidator to retrieve information about the variant based on its representation in Human Genome Variation Society (HGVS) nomenclature. Additional information includes the affected gene, genomic HGVS syntax, and a representation of the variant in Variant-Call Format (VCF)-like syntax.

```
"interpretations": [
  {
    "id": "F34 IV-3",
    "progressStatus": "SOLVED",
    "diagnosis": {
      "disease": {
        "id": "OMIM:606658",
        "label": "Spinocerebellar ataxia 15"
      },
      "genomicInterpretations": [
        {
          "subjectOrBiosampleId": "F34 IV-3",
          "interpretationStatus": "CAUSATIVE",
          "variantInterpretation": {
            "variationDescriptor": {
              "id": "var_RfhzsSQIduiweooboWpdOagcB",
              "label": "DEL423kb",
              "geneContext": {
                "valueId": "HGNC:6180",
                "symbol": "ITPR1"
              },
              "moleculeContext": "genomic",
              "structuralType": {
                "id": "SO:1000029",
                "label": "chromosomal_deletion"
              },
              "allelicState": {
                "id": "GENO:0000135",
                "label": "heterozygous"
              }
            }
          }
        }
      ]
    }
  }
],
```


Figure S2: Representation of a chromosomal deletion. The medical literature contains many case reports in which structural variants, defined here as variants that are at least 50 nucleotides in size but may extend to hundreds of thousands or millions of nucleotides, are represented only by a qualitative description. For instance, here the variant in the original publication was represented as "DEL423kb". The genotype of the variant is represented, as with smaller variants, using a term from the GENO ontology (heterozygous).
